# Protocol for 3D Bioprinting Mesenchymal Stem Cell–derived Neural Tissues Using a Fibrin-based Bioink

**DOI:** 10.21769/BioProtoc.4663

**Published:** 2023-05-05

**Authors:** Milena Restan Perez, Nadia Z Masri, Jonathan Walters-Shumka, Sarah Kahale, Stephanie M. Willerth

**Affiliations:** 1 Axolotl Biosciences, 3800 Finnerty Road, Victoria, BC, V8W 2Y2, Canada; 2 Division of Medical Sciences, University of Victoria, 3800 Finnerty Road, Victoria, BC, V8W 2Y2, Canada; 3 Department of Mechanical Engineering, University of Victoria, 3800 Finnerty Road, Victoria, BC, V8W 2Y2, Canada; 4 Centre for Advanced Materials and Technology, University of Victoria, 3800 Finnerty Road, Victoria, BC, V8W 2Y2, Canada; 5 School of Biomedical Engineering, University of British Columbia, 2222 Health Sciences Mall, Vancouver, BC, V6T 1Z3, Canada; 6 Cloud Nine Editing and Design

**Keywords:** 3D bioprinting, Stem cells, Mesenchymal stem cells, Tissue engineering, Dopaminergic neurons, Bioink

## Abstract

Three-dimensional bioprinting utilizes additive manufacturing processes that combine cells and a bioink to create living tissue models that mimic tissues found in vivo. Stem cells can regenerate and differentiate into specialized cell types, making them valuable for research concerning degenerative diseases and their potential treatments. 3D bioprinting stem cell–derived tissues have an advantage over other cell types because they can be expanded in large quantities and then differentiated to multiple cell types. Using patient-derived stem cells also enables a personalized medicine approach to the study of disease progression. In particular, mesenchymal stem cells (MSC) are an attractive cell type for bioprinting because they are easier to obtain from patients in comparison to pluripotent stem cells, and their robust characteristics make them desirable for bioprinting. Currently, both MSC bioprinting protocols and cell culturing protocols exist separately, but there is a lack of literature that combines the culturing of the cells with the bioprinting process. This protocol aims to bridge that gap by describing the bioprinting process in detail, starting with how to culture cells pre-printing, to 3D bioprinting the cells, and finally to the culturing process post-printing. Here, we outline the process of culturing MSCs to produce cells for 3D bioprinting. We also describe the process of preparing Axolotl Biosciences TissuePrint - High Viscosity (HV) and Low Viscosity (LV) bioink, the incorporation of MSCs to the bioink, setting up the BIO X and the Aspect RX1 bioprinters, and necessary computer-aided design (CAD) files. We also detail the differentiation of 2D and 3D cell cultures of MSC to dopaminergic neurons, including media preparation. We have also included the protocols for viability, immunocytochemistry, electrophysiology, and performing a dopamine enzyme-linked immunosorbent assay (ELISA), along with the statistical analysis.

Graphical overview

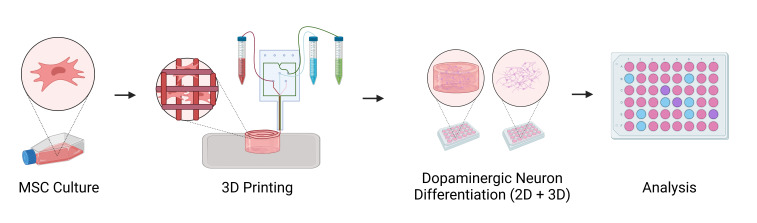

## Background

Parkinson’s disease (PD) occurs due to the depletion of dopaminergic neurons (DNs) in the substantia nigra pars compacta, which causes patients to lose their motor skills (Dauer and Przedborski, 2003). Three-dimensional bioprinting has become a sought-after technique to obtain models of such diseases, as they are low-cost and reproducible when compared to animal models, and enable a personalized medicine approach to modeling PD. 3D bioprinting deposits a cell-laden bioink in a layer-by-layer fashion using a computer-aided design (CAD) model to determine the structure with the goal of mimicking the extracellular matrix and 3D organization of cells as found in vivo (Maan et al., 2022). 3D bioprinting allows for a more accurate physiological representation of human tissues than those of 2D cell culture models. Thus, the printed models can be used as a tool for in vitro drug screening and can improve the translation between in vitro and in vivo studies (Hamadjida et al., 2019).

Bioprinting cell-laden bioinks requires large numbers of cells that must be able to withstand the stresses inherent to the bioprinting process. Mesenchymal stem cells (MSCs) derived from adipose tissue are an attractive cell type for tissue engineering as they can be obtained from patients in large numbers, while withstanding the shear stress of 3D bioprinting due to their robust characteristics (Tasnim et al., 2018). A 2011 study by Trzaska & Rameshwar differentiated bone marrow MSCs to DNs using 2D cell culture in 12 days (Trzaska and Rameshwar, 2011). In this paper, we modified the Trzaska & Rameshwar protocol by changing the sonic hedgehog to its agonist (purmorphamine) and adding both LDN-193189 (LDN) and SB431542 (SB). Bioprinted MSC-derived DN models could aid in drug discovery and enhance the chance of finding new treatments for neurological disorders like PD. In addition, bioprinted patient-derived MSC models can be used to study disease progression and to determine the most suitable treatment plan specific to each patient. Accordingly, 3D bioprinted tissue models play an important role in neural tissue engineering, especially with patient-derived stem cells that enable personalized medicine.

Human MSCs have previously been bioprinted for cartilage tissue engineering (Gao et al., 2017; Govindharaj et al., 2022). However, these articles lack detailed methodology of the bioprinting process required for other research groups to easily replicate the protocol and results. Our group has published methods describing how to make a fibrin-based bioink and how to print on the Aspect Biosystems RX1 bioprinter (Abelseth et al., 2019). However, this protocol uses stem cell–derived neural aggregates rather than a single-cell suspension, and the printing and culture parameters change depending on the cell type. Other protocols have been published on how to print with the CELLINK BIO X bioprinter, but do not explain the culture criteria and differentiation conditions for any specific cell line (Chrenek et al., 2022). Our protocol integrates several protocols into one by bridging the gap in current literature—describing how to bioprint on two different types of bioprinters, the Aspect RX1 and CELLINK BioX, how to culture MSCs before bioprinting, how to perform the preliminary cell differentiation experiments prior to bioprinting, and how to maintain and differentiate the 3D models after bioprinting. The procedures for immunocytochemistry, viability, electrophysiology, and ELISA are described in detail in this methods paper. This protocol serves as a foundation on how to bioprint other cell types such as human-induced pluripotent stem cells or a primary cell line. Although this protocol utilizes a cell-laden bioink, a cell-free scaffold can also be created using only bioink and seeding cells after the bioprinting process. Finally, this protocol describes bioprinting with an extrusion-based bioprinter, but other bioprinters could potentially be used, such as inkjet-based printers. In this case, preliminary tests without cells are required to determine the optimal printing parameters. Overall, this protocol aims to provide a standardized method for both the bioprinting of MSCs and the analysis of the bioprinted 3D tissue models.

## Materials and Reagents

1. Human adipose–derived mesenchymal stem cells (HMSC-AD) (ScienCell, catalog number: 7510); storage: liquid nitrogen
2. Phosphate-buffered saline (PBS) (Fisher Scientific, catalog number: 10010031); storage: room temperature (RT)
3. Fibronectin (ScienCell, catalog number: 8248); storage: -80 °C. Aliquot the reagent into working volumes prior to storing
4. Mesenchymal stem cell growth supplement and basal medium (complete kit) (ScienCell, catalog number: 7501); storage: -20 °C, protect from light
5. Trypan blue (0.4%) (Thermo Fisher, catalog number: 15250061); storage: RT
6. Trypsin-ethylenediaminetetraacetic acid (EDTA) (Thermo Fisher, catalog number: 25200056); storage: -20 °C. Aliquot the reagent into working volumes
7. Fetal bovine serum (FBS) (Thermo Fisher, catalog number: 12483020); storage: aliquot into working volumes and store at -20°C
8. Dulbecco’s phosphate-buffered saline (DPBS) (Thermo Fisher, catalog number: 14040117); storage: RT
9. Dimethyl sulfoxide (DMSO) (Sigma-Aldrich, catalog number: 472301); storage: RT, protect from light
10. Poly-L-ornithine (PLO) (Sigma-Aldrich, catalog number: P4957); storage: 4 °C
11. Dulbecco’s modified Eagle medium (DMEM), high glucose, no glutamine (Sigma-Aldrich, 11-960-044); storage: 2–8 °C, protect from light
12. Laminin (Sigma-Aldrich, catalog number: L2020); storage: -20 °C. Aliquot the reagent into working volumes. Avoid thaw/freeze cycles
13. Neurobasal media (Thermo Fisher, catalog number: 21103049); storage: 4 °C, protect from light
14. B-27 supplement (50×), serum free (Thermo Fisher, catalog number: 17504001); storage: -20 °C, protect from light. Aliquot the reagent into working volumes. Avoid thaw/freeze cycles
15. GlutaMAX^TM^ supplement (Thermo Fisher, catalog number: 35050061); storage: RT, protect from light
16. Penicillin-streptomycin (Sigma-Aldrich, catalog number: P4333); storage: -20 °C. Aliquot the reagent into working volumes. Avoid thaw/freeze cycles
17. Purmorphamine (Sigma-Aldrich, catalog number: SML0868); storage: -20 °C. Aliquot the reagent into working volumes. Avoid thaw/freeze cycles
18. Fibroblast growth factor 8 (FGF8) (R&D Systems, catalog number: 423-F8-025); storage: -80 °C. Aliquot the reagent into working volumes. Avoid thaw/freeze cycles
19. LDN-193189 (STEMCELL Technologies, catalog number: 72147); storage: -20 °C. Aliquot the reagent into working volumes. Avoid thaw/freeze cycles
20. SB431542 (STEMCELL Technologies, catalog number: 72232); storage: -20 °C. Aliquot the reagent into working volumes. Avoid thaw/freeze cycles
21. Brain-derived neurotrophic factor (BDNF) (PeproTech, catalog number: 450-02); storage: -20 °C. Aliquot the reagent into working volumes. Avoid thaw/freeze cycles
22. TissuePrint-HV Kit (Axolotl Bioscience); storage: -20 °C. Use for BIO X Printer
23. TissuePrint-LV Kit (Axolotl Bioscience); storage: -20 °C. Use on Aspect RX1 printer
24. TissuePrint Crosslinker (Axolotl Bioscience); storage: -20 °C. Use for both BIO X and Aspect RX1 printer
25. LIVE/DEAD Viability/Cytotoxicity kit (Thermo Fisher, catalog number: L3224); storage: -20 °C, protect from light
26. Formalin solution, neutral buffered, 10% (Sigma-Aldrich, catalog number: HT501128); storage: RT
27. Triton X-100 (Sigma-Aldrich, 9036-19-5); storage: RT
28. Normal goat serum (Abcam, catalog number: ab7481); storage: -20 °C. Aliquot the reagent into working volumes. Avoid thaw/freeze cycles
29. Primary antibody: anti-tyrosine hydroxylase (TH) antibody - neuronal marker (Abcam, catalog number: ab1120); storage: -20 °C. Aliquot reagent into working volumes. Avoid multiple freeze/thaw cycles
30. Primary antibody: anti-beta-III tubulin (TUJ1) antibody - neuron specific (R&D Systems, catalog number: MAB1195); storage: -20 °C. Aliquot reagent into working volumes. Avoid thaw/freeze cycles
31. Secondary antibody: goat anti-mouse (Alexa Fluor^®^ 488) (Thermo Fisher, catalog number: D1306); storage: 4 °C, protect from light
32. Secondary antibody: goat anti-rabbit (Alexa Fluor^®^ 568) (Thermo Fisher, catalog number: A11011); storage: 4 °C, protect from light
33. DAPI (Thermo Fisher, catalog number: D1306); storage: -20 °C (stock solution) or 4 °C (diluted solution). Avoid multiple freeze/thaw cycles
34. FLIPR Membrane Potential Assay Kit Blue (Molecular Devices, catalog number: R8042); storage: component A: RT; component B: 4 °C
35. Potassium chloride (KCl) (Caledon, catalog number: 5920170); storage: RT
36. Dopamine-specific enzyme-linked immunoassay (ELISA) kit (Abnova, catalog number: KA3838); storage: 4 °C
37. Cryovials (STEMCELL Technologies, catalog number: 200-0555)
38. T-75 flask, tissue culture treated
39. 50 mL and/or 15 mL conical, sterile
40. Various sizes of pipette tips (1,000 μL, 200 μL, 20 μL), sterile and filtered
41. Kimwipes (Fisher Scientific, catalog number: 66662)
42. Corning^®^ CoolCell^®^ containers (Cole Palmer, catalog number: RK-04392-00); storage: RT/-80 °C
43. 12-well cell culture plate (Sigma-Aldrich, catalog number: M8687)
44. LOP printhead (Aspect Biosystems, catalog number: 1256)
45. Mesh, square, 50 × 50 mm, pack of 10 (Aspect Biosystems, catalog number: 2067)
46. RX1 tubing, 74 cm (Aspect Biosystems, catalog number: 7779)
47. RX1 tubing, 54 cm (Aspect Biosystems, catalog number: 7777)
48. Empty cartridges without end and tip caps, 3 mL (CELLINK, catalog number: CSC010300502)
49. Sterile standard conical bioprinting nozzles, 22G (CELLINK, catalog number: NZ4220005001)
50. Female/female luer lock adapter (CELLINK, catalog number: OH000000010)
51. 5 cc luer lock syringe w/o needle (Terumo Medical Corporation, catalog number: SS-05L)
52. Various sizes of serological pipettes (25, 10, 5, and 1 mL)
53. Liquid nitrogen dewar
54. Control Group Cell Culture media (see Recipes)
55. Experimental Cell Culture media (see Recipes)
56. Complete Mesenchymal Stem Cell (MSC) medium (see Recipes)
57. Diluting PLO in PBS (see Recipes)
58. Diluting laminin in DMEM (see Recipes)
59. Calcein AM and Ethidium Homodimer-1 solution (see Recipes)
60. 0.1% Triton-X solution (see Recipes)
61. 5% NGS solution (see Recipes)
62. Primary Antibody solution (see Recipes)
63. Secondary Antibody solution (see Recipes)
64. 300 nM DAPI solution (see Recipes)
65. 70% ethanol solution (see Recipes)
66. Potassium chloride solution (see Recipes)
67. 10% DMSO Freezing Media solution (see Recipes)

## Equipment

1. BIO X printer (CELLINK, S-10001-001)
2. RX1 bioprinter (Aspect Biosystems, 46135)
3. DMI3000B microscope (Leica)
4. X-Cite Series 120Q Fluorescent light source (Excelitas Technologies, XI120-Q)
5. Forma^TM^ Steri-Cycle^TM^ CO_2_ incubator (Thermo Fisher, 370)
6. Infinite M200 Pro Plate Reader (TECAN, 30050303)
7. Confocal Laser scanning microscope (FIPS-Zeiss) 10× and 20× magnification
8. Retiga 2000R digital CCD camera (QImaging)
9. Biosafety cabinet (BSC)
10. Water bath (VWR, 89032-299)
11. Lab Armor^TM^ beads (Thermo Fisher, A1254301)
12. Centrifuge 5810 R (Eppendorf, 5811000015)
-80 °C freezer
14. Pipette controller
15. DeNovix CellDrop FL (DeNovix CellDrop FL)

## Software

1. Qcapture software 2.9.12
2. Excel (Microsoft)
3. ImageJ V1.52a
4. Prism 5 (GraphPad) statistical software

## Procedure

- **Culturing MSCs for cell expansion**
  Please note that this protocol has been slightly adjusted from the ScienCell protocol for the adipose-derived MSCs used in this experiment. If you obtain MSCs from a different source, it is advised that you use the specific protocol for your MSCs when expanding your cells.
  1. Coating T75 flask with fibronectin
    - Working in the BSC, add 5 mL of PBS and 150 μL of fibronectin to a 15 mL conical to make up a 30 µg/mL fibronectin solution. Mix and add to the T75 flask.
    - Gently move the flask forward and back and then side to side, to evenly coat the bottom of the plate, ensuring there are no dry spots.
    - Spray the exterior of the flask with 70% ethanol and place in the incubator at 37 °C with 5% CO_2_ overnight.
  2. Seeding cells
    - Prepare the complete MSC medium and let it come to RT (refer to Note 15). Refer to Recipe 3.
    - Remove the fibronectin-coated plate from the incubator, spray the plate with 70% ethanol, and place in the BSC.
    - Aspirate the fibronectin solution and add 15 mL of complete MSC medium to the flask. Place the flask to the side.
    - Remove a frozen cryovial of MSCs from the liquid nitrogen and place it in a 37 °C water bath without submerging the cap. Hold the cap and swirl the vial gently.
    - When a small ice crystal remains, spray the cryovial with ethanol and place it in the BSC.
    - Carefully remove the cap of the thawed cells without touching the interior threads.
    - Transfer the cells from the cryovial to the fibronectin-coated flask using a 1 mL pipette, drop by drop. Refer to Note 5.
    - Replace the cap on the flask. Move the plate forward and back and then side to side, to evenly distribute the cells.
    - Look at the cells under the microscope to ensure even distribution. Refer to Note 1.
    - Place the flask in the incubator at 37 °C and 5% CO_2_.
    - Do not disturb the plate for at least 16 h.
    - Change the media the next day and every 48 h thereafter, until the culture is approximately 50% confluent.
    - After culture is 50% confluent, perform daily media changes.
    - When culture is approximately 90% confluent, you can passage into a new plate/flask or freeze the cells.
  3. Changing media
    - Warm MSC media to RT. Refer to Note 15.
    - Retrieve T75 flask with MSCs from the incubator.
    - Check the confluency and morphology of the cells under the microscope.
    - Sterilize the plate with 70% ethanol and place in the BSC.
    - Remove the media from the flask using a 25 mL serological pipette by tilting the flask so that the media accumulates at one corner and place the serological pipette in that corner. Be careful not to scrape the bottom of the flask or touch the pipette against the mouth of the flask.
    - Add 15 mL of complete MSC media.
    - Check under the microscope to ensure that the cells are still attached to the plate surface.
    - Place the flask in the incubator at 37 °C and 5% CO_2_.
  4. Passaging cells
    - Warm complete MSC media, EDTA, FBS, and DPBS to RT.
    - Retrieve the T75 flask with MSCs from the incubator.
    - Check the confluency and morphology of the cells under the microscope to confirm the MSCs are around 90% confluent.
    - Sterilize the plate with 70% ethanol and place in the BSC.
    - Aspirate the media from the plate using a 10 mL serological pipette.
    - Gently wash cells with 5 mL of DPBS by adding DPBS and removing it shortly after.
    - Add 5 mL of 0.25% trypsin-EDTA to the flask and incubate for approximately 1–2 min, or until the cells have round up, at 37 °C. Check cells mid-way through incubation to see if they are rounding or coming off the plate.
    - Spray the flask with 70% ethanol, then place in the BSC. Transfer the trypsin solution from the flask to a 50 mL conical containing equal parts of FBS to trypsin. If 5 mL of trypsin was added to the cells, add 5 mL of FBS to the conical.
    - Place the flask back in the incubator for one more minute without any solution.
    - At the end of the incubation, gently tap the flask to detach the cells. Check under the microscope to ensure the cells have all detached.
    - Add 5 mL of FBS to the flask and transfer the contents to the 50 mL conical.
    - Examine the flask under the microscope to ensure that there is less than 5% of cells in the flask.
    - An additional wash of the flask can be performed using 5 mL of DPBS to collect any remaining cells.
    - Centrifuge at 123 *× g* for 5 min at RT.
    - Meanwhile, add 15 mL of complete MSC media to a prepared fibronectin-coated flask. Refer to step A1.
    - After centrifugation, aspirate the supernatant from the 50 mL conical. Be careful not to disturb the cell pellet.
    - Resuspend the cell pellet in 1 mL of complete MSC media and pipette up and down to ensure a homogenous cell suspension.
    - Count the cells using trypan blue and a cell counter or a hemocytometer (refer to step A5 for freezing protocol if you are not seeding the cells).
    - Plate cells at a density of 6,000 cells/cm^2^. Equation 1 can be used to calculate the quantity of cell suspension needed per well (or flask). A sample calculation for a T75 flask can be seen below. Refer to Note 6.

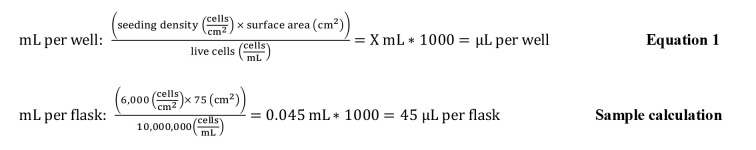

    - Add the calculated quantity of cell suspension to the flask containing 15 mL of media. Add the cells to the middle of the flask using a 1 mL pipette and move it back and forth, then side to side, to evenly distribute the cells. Refer to Note 1.
    - Check the cells under the microscope to ensure even distribution.
    - Place the flask in the incubator at 37 °C and 5% CO_2_.
    - Do not disturb the flask for at least 16 h.
    - Change the media the next day and every 48 hours thereafter, until the culture is approximately 50% confluent.
    - After culture is 50% confluent, perform daily media changes.
    - When culture is approximately 90% confluent, you can passage into a new plate or freeze the cells down.
  5. Freezing cells
    - From step A4r, after performing the cell count, add approximately 5–10 mL of complete MSC media to the remaining cell suspension.
    - Centrifuge at 123 *× g* for 5 min at RT.
    - Meanwhile, label the desired number of cryovials with the cell type, passage number, number of cells, date, and your initials. The number of cells per cryovial should be 1 million per 1 mL of 10% DMSO Freezing Media solution.
    - In a new conical, mix a 10% solution of DMSO in complete MSC media. Refer to Recipe 14.
    - After centrifugation, aspirate supernatant. Be careful not to disturb the cell pellet.
    - Add 1 mL of 10% DMSO diluted in complete MSC media per 1 million cells to the conical containing the cell pellet. Gently pipette up and down to resuspend the cells. This step must be done quickly, as cells could be adversely affected in DMSO at RT.
    - Add 1 mL of resuspended cells to each cryovial.
    - Transfer the cryovials to a controlled-rate cell freezing container and place in the -80 °C freezer for 24 h.
    - After 24 h, place cryovials in the liquid nitrogen dewar.
- **Differentiating MSCs to dopaminergic neurons on 2D cell culture plate**
  Prior to performing any bioprinting experiments, it is important to perform 2D cell culture experiments to determine if the MSCs will successfully differentiate to dopaminergic neurons and to optimize the cell density for differentiation. Please note that this protocol has been slightly adjusted from Trzaska and Rameshwar (Trzaska and Rameshwar, 2011).
  1. Coating a 12-well plate with poly-L-Ornithine/Laminin
    - Dilute PLO in PBS to reach a final concentration of 15 μg/mL. Refer to Recipe 4.
    - Add 0.5 mL of PLO/PBS per well into the 12-well cell culture plate.
    - Gently move the plate black and forth and side to side to evenly coat the bottom of the plate, ensuring there are no dry spots in the wells.
    - Wrap in parafilm and incubate at RT for 2 h. Check the plates every 30 min to ensure that it does not evaporate. Alternatively, incubate the plate overnight at 2–8 °C.
    - After 2 h or the following day, aspirate the PLO using a 10 mL serological pipette.
    - Rinse each well with 1 mL/well of PBS.
    - Rinse twice with 0.5–1 mL/well of ice-cold DMEM. Refer to Note 2.
    - In the middle of the rinses, dilute the laminin solution in DMEM to reach a final concentration of 10 μg/mL. Refer to notes section part C and D. Refer to Recipe 5.
    - Add 0.5 mL of the diluted laminin solution per well.
    - Leave for 2 h in the incubator at 37 °C and 5% CO_2_. If the plate is not being used right away, place in the fridge at 4 °C for up to one week.
    - When ready to use, aspirate the laminin and seed the cells.
  2. Seeding cells for dopaminergic differentiation
    - When the MSCs have become 90% confluent, passage them by following the step A4.
    - After performing the cell count, seed the cells onto the plate. A cell titration experiment should be performed seeding the cells at various densities to determine the optimal seeding density for your cell line. Recommendation: 1,200–25,000 cells/cm^2^. Refer to Notes 1, 6, and 7.
  3. Culturing from day -1 to day 12
    - On day -1 (the day cells are seeded), add 1 mL of complete MSC media to each well to allow cells to adhere. Perform this for both control and experimental groups. A control group is added so that it is clear that the differentiation protocol worked. Refer to Note 7.
    - Place in the incubator overnight at 37 °C and 5% CO_2_.
    - On day 0, remove the complete MSC media and add the respective media to the control group and experimental group. Refer to Recipes 1 and 2.
    - Place the dish back in the incubator for nine days without any media changes. The cells can be checked under the microscope to monitor their progress.
    - On day 9, add 50 ng/mL of BDNF directly to the experimental group without performing any media changes. Do not add the BDNF to the control group.
    - Place the dish back in the incubator and incubate for an additional three days.
    - After 12 days, the cells can be analyzed using immunocytochemistry, live/dead assays, electrophysiology, and ELISA. Refer to Figure 1 for schematic of cell culture period.
    **Figure 1.**
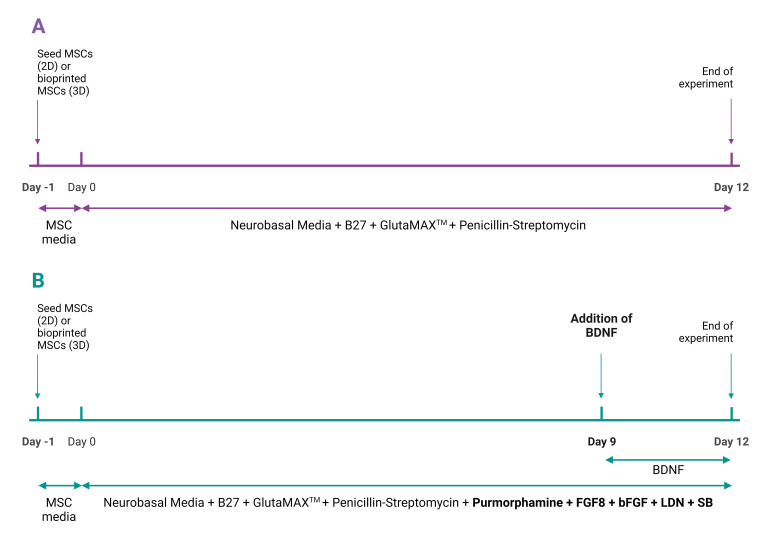
Schematic of cell culture period for (A) control group and (B) experimental group for both 2D and 3D cultures
- **Preparing the bioink**
  1. Preparing TissuePrint - Low Viscosity (LV) and High Viscosity (HV) Bioink for the Aspect Biosystem bioprinter
    - Thaw component 1 (alginate) and component 3 (fibrin) at 4 °C up to one day before use and ensure they are both homogeneous prior to using.
    - Prior to mixing the bioink, passage the MSCs in accordance with step A4.
    - Once MSCs have been counted, centrifuge the cells at 123 *× g* for 5 min. Refer to Note 8.
    - When centrifugation is completed, without disturbing the cell pellet, place the conical containing the MSCs in a conical rack and place in the incubator while the bioink components are being mixed. Do not remove the supernatant yet.
    - Thaw component 2 (genipin) at RT immediately before incorporating into the rest of the bioink.
    - Working in a sterile BSC, add component 2 to component 1 at RT. If component 2 starts to clump, slowly pipette up and down using a wide bore pipette to disperse the clump.
    - Remove the conical containing the MSCs from the incubator, disinfect the conical with 70% ethanol, and place in the BSC.
    - Carefully remove the supernatant without disturbing the cell pellet.
    - Add 1 mL of component 3 (at RT) to the cells and resuspend by pipetting up and down very gently. Harsh pipetting may cause cell death. Add the rest of component 3 to the cells and pipette up and down gently. The cell density per milliliter of bioink is dependent on the specific cell line used. Cell density titration experiments will need to be done to discover which density is best for each cell line. Typically, the cell density used is from 2 to 10 million cells per milliliter of bioink. Refer to Note 8.
    - Slowly add component 3 and MSC mixture into the mixture of component 1 and component 2 using a 1 mL pipette tip. Slowly mix the complete bioink solution by gently pipetting up and down. Avoid generating air bubbles. Refer to Note 9.
  2. Preparing Crosslinker
    - Thaw component A and component B at 4 °C.
    - Add component B to component A using a serological pipette and pipette up and down to mix.
- **CAD design**
  1. Choosing a design for the Aspect Biosystems RX1 bioprinter
    - Select “New STL” and input parameters for the structure.
    - Select “Save STL” and name your STL file.
    - Select “Infill Editor” and under Material 1, choose a rectilinear infill pattern, with 40% infill, 0 perimeters, and a fiber diameter of 0.1 mm.
    - Select “Build Design” to convert the STL file into a Design file and name your file.
  2. Choosing a design for the CELLINK BIO X bioprinter
    - Select “Bioprint” on the BIO X touchscreen.
    - Select the “3D models” tab and choose the dome model that has dimensions of 10 × 3 mm.
    - Select the “Surface” tab and select the 12-well plate and its vendor.
    - Select the “Printer” tab and choose “Tool 1” and the tool type, which should be the 3 mL Pneumatic syringe.
    - Select the “Layers” tab; for the “Grid” select rectilinear, and for the “Infill density” choose between 20%–25%. These parameters might need to be adjusted during the printing process depending on your printer.
- **Bioprinting**
  1. Bioprinting using the Aspect Biosystems RX1 bioprinter
    - The day before print day, autoclave all the equipment in Figure 2. Ensure that you have the necessary equipment for bioprinting, including the LOP printhead, RX1 tubing, and mesh sheets.
    - Refer to Video 1: Bioprinting with the RX1 Aspect Biosystem Bioprinter for a detailed explanation of how to print with this system.
    - Once the constructs have been bioprinted, use a sterile spatula to pick up the construct off the print stage, and use another spatula to carefully slide the construct off the first spatula onto the plate. The plate should be a PLO/Laminin-coated 12-well plate containing the appropriate media (one construct per well). Refer to Note 10.
    **Figure 2.**
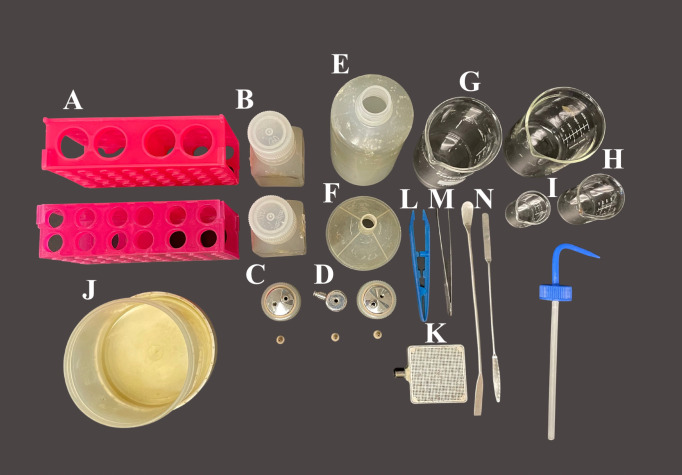
Materials to be autoclaved for 3D bioprinting with the aspect. A) Conical holders (2×); B) 125 mL Nalgene bottles (2×); C) 125 mL Nalgene bottle printing lid and tubing fastener (2×); D) 15 mL bioink conical printing lid and tubing fastener; E) 500 mL squirt bottle; F) Funnel; G) 200 mL beaker (2×); H) 50 mL beaker; I) 20 mL beaker; J) Nalgene container; K) Printing stage; L) Plastic tweezers; M) Metal tweezers; N) Spatulas (2×).
    **Video 1.**
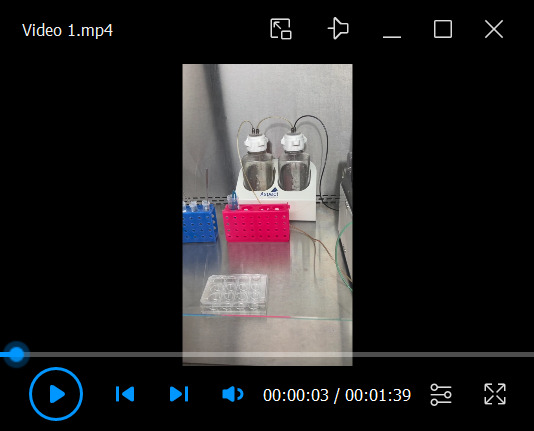
Bioprinting with the RX1 Aspect Biosystem
  2. Bioprinting using the BIO X by CELLINK
    - The day before print day, autoclave all the equipment in Figure 3.
    - Refer to Video 2: Bioprinting with the CELLINK RX1 Bioprinter for a detailed explanation of how to print with this system. Please note that the bioink has been colored with green food dye for visualization purposes.
    - Once the constructs have been bioprinted, add 1 mL of the crosslinking solution to the well. Let this sit for 2–3 min and, using a sterile spatula, carefully transfer the 3D bioprinted constructs to a PLO/Laminin-coated 12-well plate containing the appropriate media (one construct per well). Refer to Note 10.
    **Figure 3.**
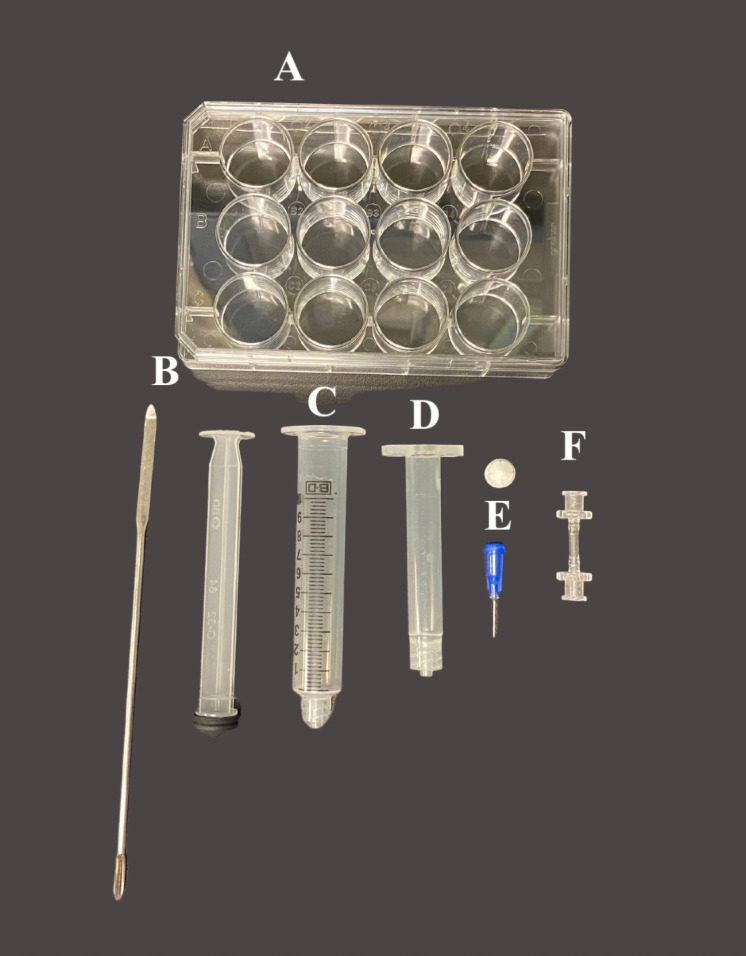
Materials needed for 3D bioprinting with the BioX. A) 12-well cell culture plate; B) Spatula; C) 5 cc luer lock syringe w/o needle; D) 3 mL cartridge; E) 22G, bioprinting nozzles and stopper; F) Female/female luer lock adapter.
    **Video 2.**
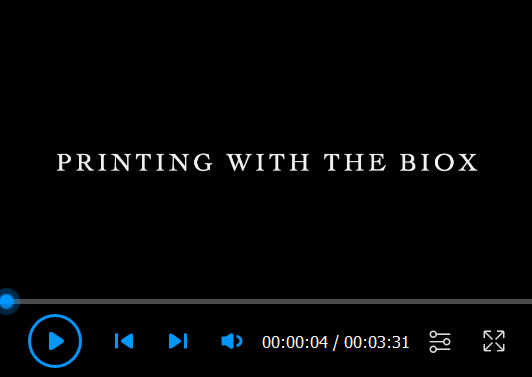
Bioprinting with the CELLINK RX1 Bioprinter
- **Culturing MSCs post-3D bioprinting**
  1. Coating plate with poly-L-ornithine/Laminin
    - Refer to Notes 1, 2, 3, 4, and 11.
    - Refer to protocol section B1 for steps on how to coat a plate with PLO/Laminin.
  2. Culturing 3D bioprinted constructs
    - On day -1 (the day cells are 3D bioprinted), add 1 mL of complete MSC media to each well to allow cells to adjust to their new environment.
    - Place in the incubator overnight at 37 °C and 5% CO_2_.
    - On day 0, remove the complete MSC media and add 2 mL (for 12-well plate) of the respective media to the control group and experimental group. Refer to recipes 1 and 2. Ensure that the constructs are fully covered by the media—if a section of the construct is sticking out, add more media as needed.
    - Place the plate back in the incubator for nine days without any media changes. The cells can be monitored under a microscope to check their progress.
    - On day 9, add 50 ng/mL of BDNF directly to the experimental group without performing any media changes. Do not add the BDNF to the control group.
    - Place the plate back in the incubator and incubate for an additional three days.
    - After 12 days, the cells can be analyzed. Refer to Figure 1 for schematic of cell culture period.
- **Cell viability**
  The viability assay was performed using the LIVE/DEAD^TM ^Viability/Cytotoxicity kit on day 9 (before adding BDNF) and on day 12.
  1. Remove the cells from the incubator and check under the microscope to see their morphology.
  2. Aspirate media from each cell culture well.
  3. Make a solution with 0.05% Calcein AM (component A) and 0.2% ethidium homodimer-1 (component B) in DPBS. Refer to Recipe 6. Determine beforehand how much complete solution you need according to how many wells you need to study. Typically, per well, 1 mL of complete solution is added. If you have 12 wells, make up a solution with a total volume of 12 mL. Make sure that the 3D construct is completely covered by the solution.
  4. Wash once with 1–2 mL of DPBS.
  5. Add the Calcein/ethidium solution to the wells to fully submerge the construct for 3D or the cells for 2D.
  6. Incubate the cells at 37 °C with 5% CO_2_ for 30 min for 3D or 15 min for 2D.
  7. Image the constructs with the Leica DMI3000B microscope with an X-Cite Series 120Q fluorescent light source using the Qcapture software 2.9.12.
  8. Quantify viability by imaging one spot on the construct and taking 15 images in equally spaced Z-planes. At each Z-plane, take one image with the 488 nm laser to visualize live cells and one image with the 543 nm laser to visualize dead cells. Ensure when saving these images that you note if it is a live or dead image.
  9. Create a Z-projection using ImageJ V1.52a software.
  10. Count the number of live and dead cells using the ImageJ software to determine the viability throughout the construct. The live and dead cells are fluorescently labeled green and red, respectively, with the LIVE/DEAD^TM ^Viability/Cytotoxicity kit. Refer to the Data analysis section part A.
- **Immunocytochemistry**
  Immunocytochemistry can be performed on both 3D and 2D cultures on day 12 to visualize the early neuronal marker TUJ1 and dopamine-specific marker tyrosine hydroxylase (TH). Refer to Note 14.
  1. Aspirate media from cell culture plate wells.
  2. Wash the cells twice with PBS.
  3. Fix the cells with 10% formalin solution and incubate at RT for 20 min for 3D culture or 10 min for 2D culture.
  4. Aspirate formalin.
  5. Wash cells twice with PBS, with 2 min incubations in between each wash, at RT.
  6. Permeabilize the cells with 0.1% Triton-X and incubate at RT for 45 min for 3D culture or 10 min for 2D culture. Refer to Recipe 7.
  7. Aspirate Triton-X solution.
  8. Block cells with 5% NGS in PBS and incubate for 1–2 h at RT. Refer to Recipe 8.
  9. Add two primary antibodies: anti-TH at 1:500 and anti-TUJ1 at 1:100 dilution in 5% NGS in PBS. Refer to Recipe 9.
  10. Incubate at 4 °C overnight with shaking at 1 × g.
  11. Wash three times with PBS after incubation.
  12. Add the secondary antibodies (goat anti-mouse Alexa Fluor^®^ 488 and goat anti-rabbit Alexa Fluor^®^ 568) at a 1:200 dilution in 5% NGS in PBS. Refer to Recipe 10.
  13. Incubate for 2 h at RT with shaking at 1 × g.
  14. Wash the cells twice with PBS.
  15. Stain the cells with 300 nM of DAPI diluted in PBS. Refer to Recipe 11.
  16. Incubate for 5 min at RT.
  17. Wash cells twice with PBS.
  18. Image the cells using the FIPS-Zeiss Confocal Laser Scanning Microscope.
  19. Following this, count the number of DAPI-positive, TUJ1-positive, and TH-positive cells using the ImageJ V1.52a software. Refer to the Data analysis section part B.
- **Electrophysiology**
  1. Turn off the lights in the BSC (the voltage-sensitive dye is light-sensitive).
  2. Aspirate 1 mL of media and leave behind 1 mL of media. For the 3D constructs, be careful not to aspirate part of the construct.
  3. Add 1 mL of FLIPR Membrane Potential Assay Kit Blue to the cells and 3D constructs so that the ratio between media and the FLIPR assay dye is 1:1.
  4. Place the plate in the incubator for 45 minutes at 37 °C with 5% CO_2_.
  5. Read the plate using a TECAN Infinite M200 Pro Plate Reader set to read fluorescent emission at 560 nm and take 25 reads from each well in a 5 × 5 grid.
  6. After the initial readings, remove 1.56 mL of the solution in the well and add KCl at a concentration of 56 mM in distilled water. Refer to Recipe 13.
  7. Incubate the 3D constructs or 2D cell cultures for 30 min at 37 °C with 5% CO_2_.
  8. After incubation, read the plates using the TECAN Infinite M200 Pro Plate Reader set to read fluorescent emission at 560 nm and take 25 reads from each well in a 5 × 5 grid.
  9. Refer to Note 12 and the Data analysis section part C.
- **Dopamine enzyme-linked immunosorbent assay (ELISA)**
  1. Collect the culture media from 3D or 2D cultures for both control and experimental groups on day 12. Collect the media from three different constructs, so that you have three replicates.
  2. Analyze the concentration of dopamine released using a dopamine-specific ELISA kit by following the manufacturer’s instructions. Refer to Note 13.
  3. Read the output absorbance using the TECAN Infinite M200 Pro Plate Reader set to 450 nm with a 635 nm reference wavelength, or as described in your chosen dopamine ELISA kit.
  4. Refer to Data analysis section part D.

## Data analysis

- **Viability**
  1. Open the live and dead images on ImageJ and create a Z project at one spot of the construct. Ensure that cells are not hiding behind other cells when doing the Z projection. If this occurs, perform the steps below on each image without performing a Z stack.
  2. Count the live and dead cells separately by following the steps below:
  3. Process > Subtract background > 12 pixels > Ok > Image > Adjust > Threshold > B&W > Adjust slider so only the cells are dark and background is white > Apply > Process > Binary > Fill Holes > Process > Binary > Convert to Mask > Process > Binary > Watershed > Analyze > Analyze Particles > 120-infinity (adjust accordingly) > Show > Outlines > Exclude on Edges > Ok.
  4. Calculate the cell viability at each section of the construct using Equation 2.
  5. Perform these calculations for three different constructs.

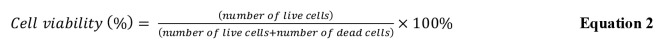

- **Immunocytochemistry**
  1. Open the images on ImageJ and apply a different color for each antibody.
  2. Create a Z stack and save these files.
  3. Create a composite image for visualization purposes.
  4. Count the number of DAPI-positive, TUJ1-positive, and TH-positive cells using ImageJ.
  5. Perform a two-tailed Student’s *t*-test, with a confidence level of 95% (p < 0.05). Perform this to determine if there is a statistically significant difference between the number of cells expressing neural cell markers to the number of nucleated cells between the control and experimental group. Perform this test on Prism 5 (GraphPad) statistical software.
- **Electrophysiology**
  1. To calculate the membrane potential, use equation 3 below, where *R* is the gas constant (8.314 J/Kmol), *T* is the temperature in Kelvin, *F* is Faraday’s constant is As/mol, *z* is the apparent charge of the external dye (assumed to be -0.64 for this experiment, but can be determined experimentally), and *∆F = F – F_o_* is the readings from the microplate reader where *F* is the reading with the constructs and cells, and *F_o_* is the background fluorescence reading of just the construct (Robinson et al., 2019). It is recommended that these calculations are performed on Excel.

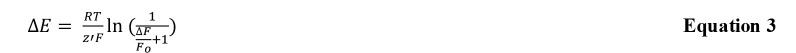

  2. Using Prism 5 (GraphPad) statistical software, perform a one-way ANOVA and Tukey post-hoc analysis using a confidence level of 95% (p < 0.05).
- **Dopamine ELISA**
  1. Obtain a standard curve by plotting the mean absorbance readings on the y-axis (linear) against the corresponding standard concentrations (logarithmic) on the x-axis.
  2. Obtain a spline curve fit using Prism 5 statistical software and interpolate the unknown concentration values for each unpaired absorbance reading.
  3. This assay is a competitive assay, so as absorbance values decrease, the concentration increases.

## Notes

1. Always label plates with either your full name or initials, the date (Month/Day/Year), what the plate contains, and cell passage number.
2. PLO is toxic to cells, so the rinsing steps are critical.
3. Always ensure laminin is cold. Do not let laminin come to RT. Avoid freeze/thaw cycles—laminin should not be frozen more than twice.
4. Laminin should be aliquoted prior to use. The volume and concentration of laminin varies from batch to batch. Always refer to the batch number before aliquoting.
5. Dilution and centrifugation of the cells after thawing is not recommended, since these actions are more harmful to the cells than the effect of residual DMSO in the culture. Therefore, add the cells directly to the media in the new cell culture plate and change the media the next day to remove any DMSO once the cells are attached.
6. The number of live cells is what you will be using to do your calculations for the number of cells that will be added to the wells. Do not use the total number of cells.
7. Seed enough wells so that you can have three replicates for each cell density and control groups.
8. Ensure that you have enough MSCs for your printing purposes. The seeding density can vary from 2 to 10 million cells per milliliter of bioink, depending on your cell line. This may require multiple weeks of passaging MSCs so that you can accumulate enough cells.
9. For best results, use within one hour. It is also recommended that small batches of bioink are made (approximately 4 mL at a time), so that if bioprinting experiences any setbacks, materials and cells are not wasted.
10. It is important that you prepare the PLO/Laminin plate a day before bioprinting, so that plates are ready during the bioprinting process.
11. Perform this step a day or two before printing, so that plates are ready for print day.
12. We used this method as 3D tissues cannot be easily patch-clamped. This analysis is performed on both control and experimental groups. It is also performed on cell-free constructs (3D) or blank wells (2D), which are a control for background fluorescence.
13. Our lab used the Abnova dopamine ELISA kit (refer to Materials and Reagents section for catalog number) because it could detect low concentrations of dopamine.
14. A titration of the primary and secondary antibodies is recommended, to determine the most optimal concentration of each antibody for your cell line.
15. It is not recommended to place the media in a water bath, as this can cause some components to degrade.

## Recipes

1. **Control Group Cell Culture Media**
  Neurobasal Media (NBM)
  2% B-27 supplement
  1% GlutaMAX^TM^
  1% Penicillin-streptomycin
2. **Experimental Cell Culture Media**
  Control Group Cell Culture Media
  250 ng/mL purmorphamine
  100 ng/mL of FGF8
  100 nM of LDN-193189
  10 µM of SB431542
3. **Complete Mesenchymal Stem Cell (MSC) Medium**
  Mix all components of the MSC media kit, which include:
  500 mL of MSC basal medium
  25 mL of fetal bovine serum
  5 mL of mesenchymal stem cell growth supplement
  5 mL of penicillin/streptomycin solution
4. **Diluting PLO in PBS**
  - Depending on the lot number of your PLO, obtain the certificate of analysis (COA) from the Sigma-Aldrich website and determine the concentration of the purchased bottle.
  - Calculate the amount of PLO needed for a final volume of 6 mL (enough for a 6- or 12-well plate) using equation 4, where C_1_ is the stock concentration of PLO.
  - Add (6 - V_1_) mL of PBS to a sterile 15 mL conical.
  - Add V_1_ mL of PLO to the 15 mL conical.

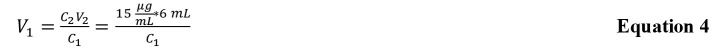

5. **Diluting Laminin in DMEM**
  - Depending on the lot number of your laminin, obtain the COA from the Sigma-Aldrich website and determine the concentration of the purchased bottle.
  - Calculate the amount of laminin needed for a final volume of 6 mL (enough for a 6- or 12-well plate) using equation 5, where C_1_ is the stock concentration of PLO.
  - Add (6 – V_1_) mL of DMEM to a sterile 15 mL conical.
  - Add V_1_ mL of PLO to the 15 mL conical.

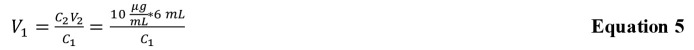

6. **Making a Calcein AM and Ethidium Homodimer-1 solution**
  - To make a 12 mL solution, add 11.97 mL of DPBS to a 15 mL conical.
  - To the same conical, add 6 µL of Calcein AM and 24 µL of ethidium homodimer-1.
7. **Making a 0.1% Triton-X solution**
  - To make a 25 mL solution of 0.1% Triton-X, add 24.975 mL of PBS to a 50 mL conical.
  - To the same conical, add 25 µL of 100% Triton X-100.
8. **Making a 5% NGS solution**
  - To make a 25 mL solution of 0.1% Triton-X, add 24.975 mL of PBS to a 50 mL conical.
  - To the same conical, add 25 µL of 100% Triton X-100.
9. **Making the Primary Antibody solution**
  - To make a 12 mL solution, add 11.856 mL of the above 5% NGS solution to a 15 mL conical.
  - To the same conical, add 120 µL of anti-TUJ1 and 24 µL of anti-TH.
  - Refer to Note 14.
10. **Making the Secondary Antibody solution**
  - To make a 12 mL solution, add 11.880 mL of the above 5% NGS solution to a 15 mL conical.
  - To the same conical, add 60 µL of Alexa Fluor® 488 and 60 µL of Alexa Fluor^®^ 568.
  - Refer to Note 14.
11. **Making a 300 nM DAPI solution**
  - Add 2 mL of distilled water to the entire contents of the DAPI vial to make a 14.3 mM DAPI stock solution.
  - In a 1 mL Eppendorf tube, add 100 µL of PBS and 2.1 µL of the 14.3 mM DAPI stock solution to make a 300 µM DAPI solution.
  - Dilute the 300 µM DAPI intermediate dilution 1:1,000 in PBS as needed to make a 300 nM DAPI stain solution.
12. **Making a 70% Ethanol solution**
  - Add 4 L of 95% ethanol to a large container.
  - Add 1.43 L of distilled water to the same container.
13. **Making a Potassium Chloride solution**
  - Add 149.1 mg of KCl powder to 20 mL of distilled water to make a 100 mM solution.
  - Mix 560 µL of the 100 mM KCl solution to 440 µL of media to make a 56 mM solution of KCl.
14. **Making a 10% DMSO Freezing Media solution**
  - To make a 10 mL solution of 10% DMSO, add 9 mL of complete MSC media to a 15 mL conical.
  - To the same conical, add 1 mL of sterile 100% DMSO.
